# Protective effects of Crocin on hepatic steatosis, oxidative stress, and lipid profile in an animal model of non-alcoholic fatty liver disease

**DOI:** 10.1371/journal.pone.0357768

**Published:** 2026-10-01

**Authors:** Hossain Rezayee, Mohammad Ali Fahmidehkar, Farimah Beheshti, Mohsen Ghoryani, Yaser Yousefpoor, Dawood Hossaini, Davod Ilbeigi

**Affiliations:** 1 Student Research Committee, Torbat Heydariyeh University of Medical Sciences, Torbat Heydariyeh, Iran; 2 Department of Paracilinic, Faculty of Medicine, Khatam Al-Nabieen University, Kabul, Afghanistan; 3 Department of Clinical Biochemistry, School of Medicine, Torbat Heydariyeh University of Medical Sciences, Torbat Heydariyeh, Iran; 4 Research Center of Advanced Technologies in Medicine, Torbat Heydariyeh University of Medical Sciences, Torbat Heydariyeh, Iran; 5 Departments of Physiology, School of Medicine, Torbat Heydariyeh University of Medical Sciences, Torbat Heydariyeh, Iran; 6 Neuroscience Research Center, Torbat Heydariyeh University of Medical Sciences, Torbat Heydariyeh, Iran; 7 Department of Laboratory Sciences, School of Paramedical Sciences, Torbat Heydariyeh University of Medical Sciences, Torbat Heydariyeh, Iran; 8 Department of Medical Biotechnology, School of Paramedical Sciences, Torbat Heydariyeh University of Medical Sciences, Torbat Heydariyeh, Iran; University of Toledo College of Medicine: The University of Toledo College of Medicine and Life Sciences, UNITED STATES OF AMERICA

## Abstract

**Objective:**

Non-alcoholic fatty liver disease (NAFLD) is the most prevalent chronic liver disorder worldwide. This study aims to investigate the effects of crocin on lipid profile, hepatic lipid accumulation, and antioxidant status in a rat model of non-alcoholic fatty liver induced by a high-fat diet.

**Materials and methods:**

Forty-two male Wistar rats were divided into six groups, each containing seven rats. Fatty liver was induced by feeding a high-fat diet for eight weeks. Subsequently, the treatment groups received intraperitoneal crocin for four weeks. The groups were then evaluated for changes in serum lipid profiles, liver function enzymes in serum, oxidative stress biomarkers and histopathological alterations in liver tissue.

**Results:**

High-fat diet feeding resulted in significant hepatic lipid accumulation, elevated serum aspartate aminotransferase, alanine aminotransferase, and alkaline phosphatase levels, increased hepatic malondialdehyde and nitrite concentrations, and impaired antioxidant enzyme activities. Histological analysis demonstrated hepatocellular steatosis, inflammatory infiltration, and hepatic structural alterations. Crocin treatment significantly attenuated lipid accumulation, normalized serum liver enzyme levels, reduced oxidative stress markers, and enhanced antioxidant enzyme activities. Histopathological evaluation further confirmed marked improvement in liver tissue morphology.

**Conclusion:**

The results of the present study demonstrate that Crocin exerts protective effects against NAFLD, highlighting its potential as a therapeutic agent for diet-induced fatty liver disease.

## Introduction

Non-alcoholic fatty liver disease (NAFLD) has become one of the most common chronic liver disorders worldwide [1,2]. According to the World Health Organization (WHO) and recent global epidemiological studies, the global prevalence of NAFLD has been estimated at approximately 24–32%, and the disease is increasingly recognized as an important cause of cirrhosis, hepatocellular carcinoma, and end-stage liver disease [3,4]. NAFLD is a progressive condition characterized by the accumulation of lipids in the liver in the absence of excessive alcohol consumption [5]. It is currently the leading cause of abnormal liver function in the United States and Europe [6]. The prevalence of NAFLD varies by region: 32% in the Middle East, 30% in South America, 27% in Asia, 24% in North America, 24% in Europe, and 13% in Africa [7,8]. It is estimated that the burden of end-stage liver disease will increase two- to threefold in Western and Asian countries by 2030 [9,10]. NAFLD is closely associated with obesity, type 2 diabetes, and other metabolic disorders, which contribute to abnormal lipid accumulation in the liver, cirrhosis, and hepatocellular carcinoma worldwide [11]. Obesity is common among NAFLD patients; approximately 75% of patients are overweight, and 90%−95% of obese individuals have NAFLD [12]. The prevalence of obesity, particularly truncal obesity, is a more significant determinant of NAFLD risk than body mass index (BMI) alone [12].

Some patients may experience only an increase in fat within the liver tissue (simple steatosis), while in others, the condition progresses to non-alcoholic steatohepatitis (NASH) [13]. As collagen progressively deposits and blood vessels regenerate, liver cirrhosis may develop [14]. Fatty liver occurs when the influx and lipogenesis of lipids exceed their oxidation and export from the liver. Insulin resistance plays a key role in the pathogenesis of NAFLD by disrupting the balance between lipid accumulation and lipid oxidation and export. Insulin resistance increases peripheral lipolysis, resulting in a higher influx of free fatty acids into the liver, which contributes to hepatic triglyceride synthesis. Furthermore, insulin resistance is a driving factor in the progression of NAFLD, ultimately leading to non-alcoholic steatohepatitis [15]. NAFLD is characterized by the accumulation of lipids—mainly triacylglycerol—exceeding 5–10% of the liver’s total weight [16]. Dietary fat intake, increased import of metabolites derived from lipolysis in white adipose tissue, and fatty acids synthesized within the liver via de novo lipogenesis are the primary sources of fat accumulation in the liver [17]. Glucose promotes hepatic lipogenesis by activating carbohydrate-responsive element-binding protein. Hepatic triglycerides are typically transported into the bloodstream as very low-density lipoproteins (VLDL) with the assistance of apolipoprotein B (APOB); disruptions in APOB can contribute to hepatic steatosis [18]. The pathogenesis of NAFLD is often explained by the two-hit hypothesis. The first hit involves the accumulation of triglycerides and free fatty acids as fat droplets exceeding 5% in liver cells, a process to which insulin resistance contributes. This accumulation predisposes the liver to inflammation, making it vulnerable to the second hit. The second hit involves oxidative stress, which causes mitochondrial dysfunction and the generation of free radicals, leading to the production of pro-inflammatory cytokines and apoptosis [5,19].

Regarding the treatment of NAFLD, the most commonly recommended therapy for obese patients is diet and physical activity. However, not everyone manages to achieve a healthy BMI through these measures alone. Therefore, it is sometimes necessary to introduce anti-obesity drugs such as Orlistat. Nevertheless, long-term use of these drugs is associated with adverse side effects, including gastrointestinal complications, decreased high-density lipoprotein (HDL) levels, and increased blood pressure [20,21]. Herbal medicines, as natural products, offer a promising alternative for the management and treatment of NAFLD [22]. One well-known and effective medicinal plant is saffron (Crocus sativus L.). This herbaceous, perennial plant belongs to the Iridaceae family, which comprises about 90 species. Known as the king of medicines, is native to Southwest Asia [23]. Today, saffron is primarily cultivated in Afghanistan, Iran, Greece, Spain, Italy, India, and several other countries [24]. Saffron contains three main components: crocin, picrocrocin, and safranal. These compounds are responsible for saffron’s characteristic color, taste, and aroma [25]. Other phytochemical constituents of saffron include various volatile compounds (more than 150), non-volatile compounds such as carotenoids, lycopene, and α- and β-carotenes, as well as aromatic compounds and vitamins like riboflavin and thiamine [24]. Crocin is a carotenoid compound [26] that exhibits a wide range of biological activities, including anti-genotoxic, cytotoxic, antioxidant, anti-atherosclerotic, hypolipidemic, hypoglycemic [27], anti-inflammatory, anti-cancer [28], neuroprotective, antihypertensive, anti-obesity [29], promotion of fat cell differentiation and lipolysis [30], and reduction of serum lipid levels [31]. Crocin inhibits activated AMP-activated protein kinase (AMPK), lipolysis, and acetyl-CoA carboxylase—an important enzyme for lipid synthesis—during adipogenesis. Moreover, it suppresses the expression of sterol regulatory element-binding protein-1 (SREBP-1), C/EBPα, PPARγ, and fatty acid synthase (FAS), while increasing beta-oxidation of fatty acids, thereby improving fatty liver and metabolic disorders [32]. The aim of this study is to investigate the effect of crocin on disorders induced by NAFLD.

## Materials and methods

### Animals

A total of 42 healthy, 6-week-old male Wistar rats (180–200 g) were obtained from the animal house of Torbat Heydariyeh University of Medical Sciences. All rats were housed in Plexiglas cages (3 rats per cage) with dimensions of 40 × 25 × 20 cm and maintained at a stable room temperature (22 ± 2 °C) with a 12-hour light/dark cycle (lights on at 7:00 AM), with free access to food and water. All experimental procedures were approved by the Institutional Animal Care and use Committee of Torbat Heydariyeh University of Medical Sciences. The Sample size (n = 7 per group) was determined based on previously published experimental NAFLD animal studies and ethical considerations for animal research [33]. The present study was conducted and reported in accordance with the ARRIVE guidelines.

### Induction of obesity and experimental design

Following a one-week acclimatization period, forty-two male Wistar rats were randomly assigned to receive either a normal diet (ND) or a high-fat diet (HFD) for 8 weeks to induce non-alcoholic fatty liver disease (NAFLD). After completion of the obesity induction period, the animals were randomly divided into six experimental groups (n = 7 per group): Group I (control): normal diet (ND) + saline treatment; Group II (Fat): high-fat diet (HFD) + saline treatment; Group III: HFD + Crocin 10 mg/kg (Sigma-Aldrich, USA; catalog no. 17304); Group IV: HFD + Crocin 30 mg/kg; Group V: HFD + Crocin 50 mg/kg; Group VI: HFD + Orlistat 10 mg/kg (Porsina, Tehran, Iran).

During both the NAFLD induction and treatment periods, the ND group received a normal diet, whereas all other groups were maintained on a high-fat diet throughout the study. After the 8-week dietary induction phase, a 4-week treatment period was initiated. During this phase, Groups I and II received intraperitoneal injections of normal saline as vehicle once daily. Rats in Groups III, IV, and V were administered crocin intraperitoneally at doses of 10, 30, and 50 mg/kg/day, respectively [34,35]. Group VI received intraperitoneal Orlistat at a dose of 10 mg/kg/day. Crocin and Orlistat were dissolved in normal saline, and all treatments were administered once daily for 4 consecutive weeks.

After acclimatization, rats were fed either a normal diet (ND; control group) or a high-fat diet (HFD) for 8 weeks. The HFD consisted of approximately 60% kcal from fat, 20% from carbohydrates, and 20% from protein, while the ND provided approximately 10% kcal from fat. Both experimental diets were purchased from Zist Fanavaran Royan Imen Co. (Tehran, Iran). The detailed composition of the experimental diets is presented in Table 1. Following 8 weeks of obesity induction, the rats were divided into six groups (n = 7 each): Group I: normal diet (ND); Group II: high-fat diet (HFD); Group III: HFD + crocin 10 mg/kg; Group IV: HFD + crocin 30 mg/kg; Group V: HFD + crocin 50 mg/kg; Group VI: HFD + Orlistat 10 mg/kg. The ND Group was fed a normal diet, while the other groups were fed a high-fat diet during treatment. Then, the ND and HFD groups received intraperitoneal injections of normal saline, while the other groups received intraperitoneal injections of crocin or Orlistat for 4 weeks. Three different doses of crocin (10, 30, and 50 mg/kg/day) were administered intraperitoneally to the III, IV and V groups and VI group was injected intraperitoneally with Orlistat at a dose of 10 mg/kg for 4 weeks.

**Table 1:** Composition of the normal diet (ND) and high-fat diet (HFD) used in this study.

Ingredients	Normal Diet (g/kg diet)	High-Fat Diet (g/kg diet)
**Corn starch**	650	150
**Casein**	200	200
**Beef tallow**	0	400
**Corn oil**	50	0
**Sucrose**	0	150
**Cellulose**	50	50
**Mineral premix**	35	35
**Vitamin premix**	10	10
**Methionine**	3	3
**Choline bitartrate**	2	2

Data are expressed as g/kg diet. The ND provided approximately 10% of calories from fat, whereas the HFD provided approximately 60% of calories from fat.

### Blood collection and tissue preparation

At the end of the treatment period, following a 12-hour overnight fast, the rats were deeply anesthetized using CO_2_ inhalation to minimize suffering. Blood samples (approximately 5–7 mL) were then collected via carotid artery puncture. The animals were subsequently euthanized, and liver tissues were excised for subsequent biochemical and histopathological analyses. Blood was collected into clean, dry centrifuge tubes, and plasma was separated by centrifugation at 3000 rpm (Sigma Centrifuge, Germany; Model 3–18KS) for 15 minutes. Plasma samples were stored frozen for biochemical analyses, including triglycerides (TG), total cholesterol (TC), high-density lipoprotein (HDL), low-density lipoprotein (LDL) cholesterol, and oxidative stress parameters. Subsequently, the rats underwent surgery, and liver tissue was collected for histological studies.

### Assessment of serum lipid profile

Serum triglycerides, total cholesterol, high-density lipoprotein cholesterol, and low-density lipoprotein cholesterol were measured using enzymatic colorimetric assays with commercial kits (Pars Azmoon, Tehran, Iran) according to the manufacturer’s instructions. Triglycerides were determined using the glycerol-3-phosphate oxidase–phenol aminophenazone (GPO-PAP) method, and total cholesterol was measured using the cholesterol oxidase–phenol aminophenazone (CHOD-PAP) method. HDL-C and LDL-C concentrations were determined using enzymatic cholesterol esterase/cholesterol oxidase (CHE/CHO) colorimetric methods [36]. All measurements were performed using an automated clinical chemistry analyzer (Mindray, China; Model BS-380) under standardized laboratory conditions.

### Liver function enzymes assay

Serum aspartate aminotransferase (AST) and alanine aminotransferase (ALT) activities were determined using a colorimetric method based on the Reitman–Frankel procedure [37]. Alkaline phosphatase (ALP) activity was measured using a colorimetric method according to the Bessey–Lowry method [38]. All analyses were performed using commercial diagnostic kits (Pars Azmoon, Tehran, Iran) on an automated clinical chemistry analyzer (Mindray, China; Model BS-380) according to the manufacturer’s instructions [39].

### Evaluation of oxidative stress markers (MDA, total thiol and nitrite) in liver tissue

To assess the extent of lipid peroxidation, the levels of malondialdehyde (MDA) were measured using the thiobarbituric acid (TBA) reaction. This assay involved the reaction of MDA with TBA (Merck, Germany; catalog no. 1081800025), trichloroacetic acid (Sigma-Aldrich, USA; catalog no. T6399), and hydrochloric acid (Shimaraz, Iran; catalog no. 7647-01-0). The final oxidation products of unsaturated fatty acids with MDA yield a red complex, which is detected at 532 nm using a Jenway spectrophotometer (United Kingdom; Model 6105). Concurrently, thiol content, a recognized mediator of the antioxidant defense system, was measured according to Ellman’s instructions. This method relies on the formation of a yellow complex resulting from the reaction between thiol groups and DTNB (2,2’-dinitro-5,5’-dithiobenzoic acid) (Sigma-Aldrich, USA; catalog no. D8130), with detection at 412 nm [32]. The nitrite levels were assessed using the Griess reagent method (Promega kit, USA; catalog no. G2930). Briefly, 100 μL of supernatant was added to the Griess reagent. The mixture was then transferred to a microplate, and the absorbance was measured at 520 nm (Hyperion ELISA reader, USA; Model MPR4+). The final nitrite concentration was calculated from a standard calibration curve [40].

### Measurement of antioxidant enzyme activities

SOD and CAT as two major antioxidant enzymes, were measured using colorimetric techniques. Briefly, one unit of SOD activity was defined as the amount of enzyme that inhibits the reduction rate of MTT (3-(4,5-dimethylthiazol-2-yl)-2,5-diphenyltetrazolium bromide; Sigma Aldrich, USA; catalog no. 475989) by 50%. Furthermore, the Aebi protocol was employed to determine CAT activity, which involves the reduction of hydrogen peroxide to water and oxygen. The absorbance for SOD and catalase was measured at 420 nm and 240 nm, respectively [32].

### Histopathological analysis

For tissue examination, the liver was removed from the abdominal cavity and rinsed with physiological saline solution. It was then fixed in 10% formaldehyde (Merck, Germany; catalog no. K47267802019) for 24 h. Liver tissue samples were processed with tissue processor (Leica Biosystems, Germany; Model TP1020), embedded in paraffin blocks and 5-micron sections were prepared (Leitz microtome, Germany, Model 1512). Then, Sections were deparaffinized in xylene (Merck, Germany; catalog no. 108634) for 20 min and stained using the hematoxylin-eosin staining method (Kalazist, Iran; catalog no. 104302–109253). A total of 42 pathology slides were prepared, corresponding to the number of study groups. Subsequently, 10 microscopic fields from each slide were photographed at 400x magnification using an Olympus CX21FS1 optical microscope (Japan) equipped with a camera. The pathological evaluation was conducted independently and in a double-blind manner by two pathologists to ensure the accuracy and objectivity of the findings [41]. Histopathological changes were assessed semi-quantitatively based on steatosis, inflammation, ballooning and histopathological changes. Steatosis was scored as 0 = absent, 1 = mild, 2 = moderate, and 3 = severe. Inflammation, ballooning and histopathological changes were also scored according to severity [42].

### Statistical analysis

Data are presented as mean ± standard deviation (SD). Statistical analyses were performed using SPSS version 16. One-way analysis of variance (ANOVA) was conducted to compare groups, followed by Tukey’s post hoc test for pairwise comparisons. Differences were considered statistically significant at p < 0.05.

### Ethical approval

The study was approved by the Ethical Committee of Torbat Heydariyeh University of Medical Sciences (IR.THUMS.AEC.1401.003).

## Results

### Effect of crocin on lipid profile of rats

The effects of crocin on the TG, TC, LDL-C, and HDL-C in plasma in each group are shown in Fig 1. The high-fat diet group exhibited a significant increase in the mean serum levels of TG, TC, and LDL-C compared to the control group (p = 0.0008, p = 0.003 and p = 0.0006 respectively), attributable to the high-fat diet. The results demonstrate that a high dose of crocin (Cr 50 mg/dl) significantly reduced the mean levels of TG and LDL-C (p = 0.009 and p = 0.026 respectively).

**Fig 1:**
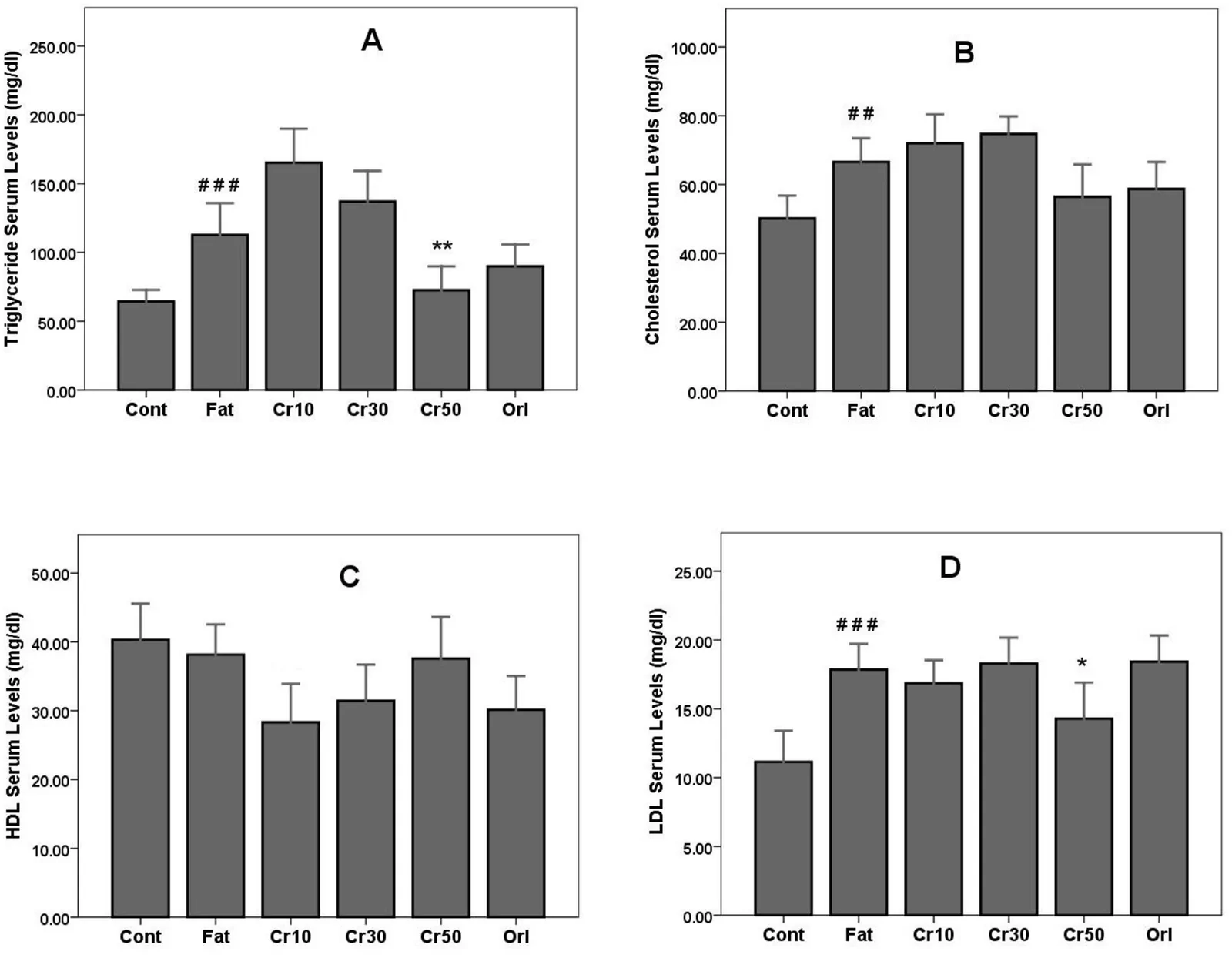
The effect of different doses of crocin and orlistat on lipid profile in the serum of the studied groups. Serum concentrations of (A) triglycerides (TG), (B) total cholesterol (TC), (C) high-density lipoprotein cholesterol (HDL-C), and (D) low-density lipoprotein cholesterol (LDL-C) were evaluated. Experimental groups (n = 7 per group) were defined as follows: ND, normal diet (control group); HFD, high-fat diet (Fat) group; Cr10, Cr30, and Cr50, animals receiving crocin at doses of 10, 30, and 50 mg/kg body weight, respectively; Orl, animals treated with orlistat. Data are presented as mean ± SD. ^##^ p < 0.01, ^###^p < 0.001 compared to the group receiving ND. *p < 0.05, **p < 0.01 and ***p < 0.001 compared to the HFD.

### Effect of crocin on liver enzymes of rats

Changes in liver enzyme levels in rats fed a high-fat diet and treated with crocin are shown in Fig 2A–C. The fat group exhibited a significant increase in serum ALT, AST, and ALP levels compared to the ND group (p = .002, p = 0.0004 and p = 0.0009 respectively). Conversely, treatment with crocin at 30 mg/kg significantly decreased AST levels (P = 0.00002) and crocin 50 mg/kg significantly reduced serum AST and ALP levels (P = 0.00003 and P = 0.00002, respectively). Additionally, Orlistat significantly reduced serum ALT, AST and ALP levels (p = 0.006, p = 0.001 and p = 0.0001 respectively).

**Fig 2:**
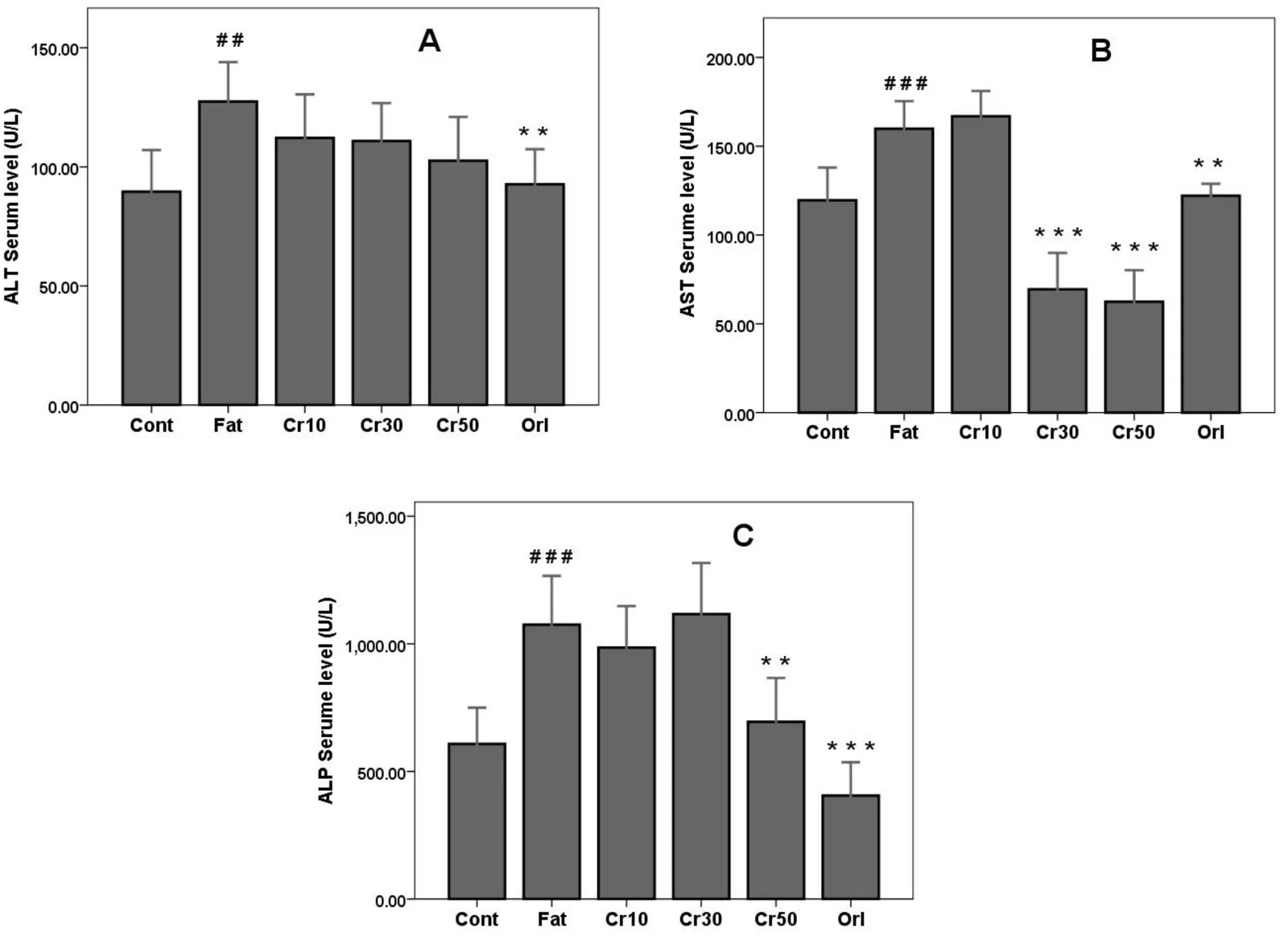
The effect of different doses of crocin and orlistat administration on serum liver enzyme levels in the studied groups. Serum activities of (A) Alanine Aminotransferase (ALT), (B) Aspartate Aminotransferase (AST), and (C) Alkaline Phosphatase (ALP) were measured as biochemical indicators of liver function. Experimental groups (n = 7 per group) were defined as follows: ND, normal diet (control group); HFD, high-fat diet (Fat) group; Cr10, Cr30, and Cr50, animals receiving crocin at doses of 10, 30, and 50 mg/kg body weight, respectively; Orl, animals treated with orlistat. Data are presented as mean ± SD. ^##^ p < 0.01, ^###^p < 0.001 compared to the group receiving ND. *p < 0.05, **p < 0.01 and ***p < 0.001 compared to the HFD.

### Effect of crocin on oxidative stress in rats

Biochemical analysis of liver tissue indicated that a HFD significantly increased MDA and nitrite concentrations (p = 0.00004 and p = 0.00002 respectively; Fig 3A and E), decreased total thiol concentration (p = 0.00008; Fig 3B), and suppressed the enzymatic activities of SOD and CAT (p = 0.0003 and p = 0.00007 respectively; Fig 3C and D). Intraperitoneal injections of crocin at doses of 30 mg/kg significantly reduced MDA and nitrite levels (p = 0.02 and p = 0.002 respectively; Fig 3A and E). Also, intraperitoneal injections of crocin at doses of 50 mg/kg significantly reduced MDA and nitrite levels (p = 0.0002; Fig 3A and E). Additionally, Orlistat at 10 mg/kg significantly reduced MDA and nitrite levels (p = 0.0003 and p = 0.0001 respectively; Fig 3A and E).

**Fig 3:**
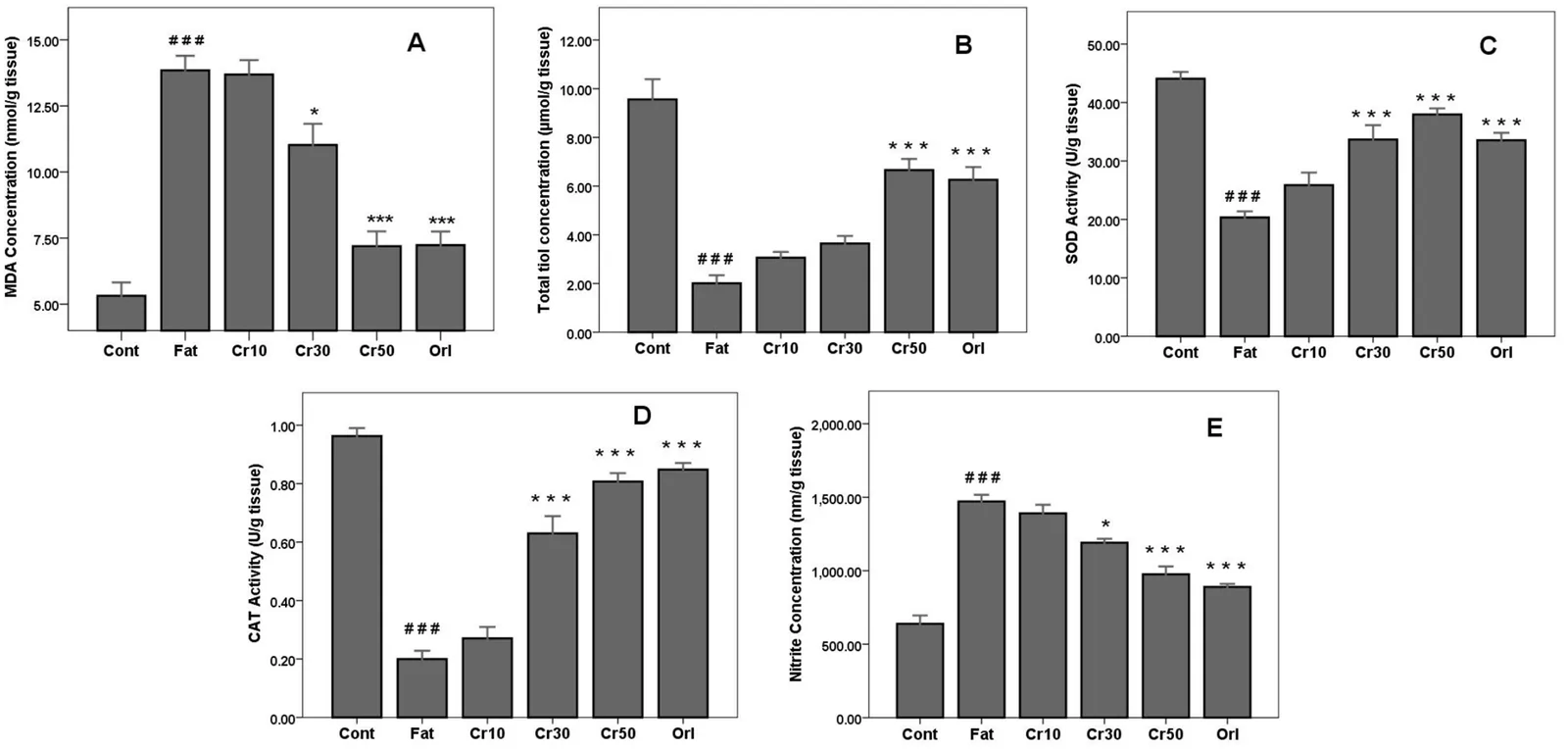
The effect of different doses of crocin and orlistat on hepatic oxidative stress parameters in the studied groups. Liver tissue levels of (A) malondialdehyde (MDA), (B) total thiol content, (C) superoxide dismutase (SOD) activity, (D) catalase (CAT) activity, and (E) nitrite were determined. Experimental groups (n = 7 per group) were defined as follows: ND, normal diet (control group); HFD, high-fat diet (Fat) group; Cr10, Cr30, and Cr50, animals receiving crocin at doses of 10, 30, and 50 mg/kg body weight, respectively; Orl, animals treated with orlistat. Data are presented as mean ± SD. ^##^ p < 0.01, ^###^p < 0.001 compared to the group receiving ND. *p < 0.05, **p < 0.01 and ***p < 0.001 compared to the HFD.

Regarding total thiol concentration, only crocin at 50 mg/kg and Orlistat at 10 mg/kg increased mentioned parameters (p = 0.0001 and p = 0.0006 respectively; Fig 3B).

The activities of SOD and CAT were significantly enhanced by crocin 30 mg/kg (p = 0.0002 and p = 0.0003 respectively) and also enhanced by crocin 50 mg/kg (p = 0.0006 and p = 0.00001 respectively) (Fig 3C and D). Additionally, the activities of SOD and CAT were significantly enhanced by Orlistat (10 mg/kg) (p = 0.0002 and p = 0.00003; Fig 3C and D).

### Histopathological assessment

Histopathological examination of liver tissue was performed based on lipid accumulation, ballooning, inflammation, and overall histopathological changes, as illustrated in Fig 4 and summarized in the semi-quantitative scoring in Table 2. The ND group exhibited a normal hepatic structure, with a score of 0 for all parameters (Fig 4A). In contrast, the HFD group displayed severe histopathological alterations, characterized by scores of 3 across all assessed parameters (Fig 4B and Fig 4B1). Treatment with crocin at 10 mg/kg resulted in minimal changes, with scores of 3, 2, 3, and 3 for lipid accumulation, ballooning, inflammation, and overall histopathological changes, respectively (Fig 4C and Fig 4C1). Crocin at 30 mg/kg attenuated these alterations, resulting in scores of 2, 1, 1, and 1, respectively (Fig 4D). Administration of crocin at 50 mg/kg resulted in a near-normal hepatic structure, with scores of 1 for lipid accumulation and 0 for the remaining parameters (Fig 4E). Finally, the orlistat 10 mg/kg displayed a normal hepatic structure, scoring 0 for all evaluated parameters (Fig 4F).

**Fig 4:**
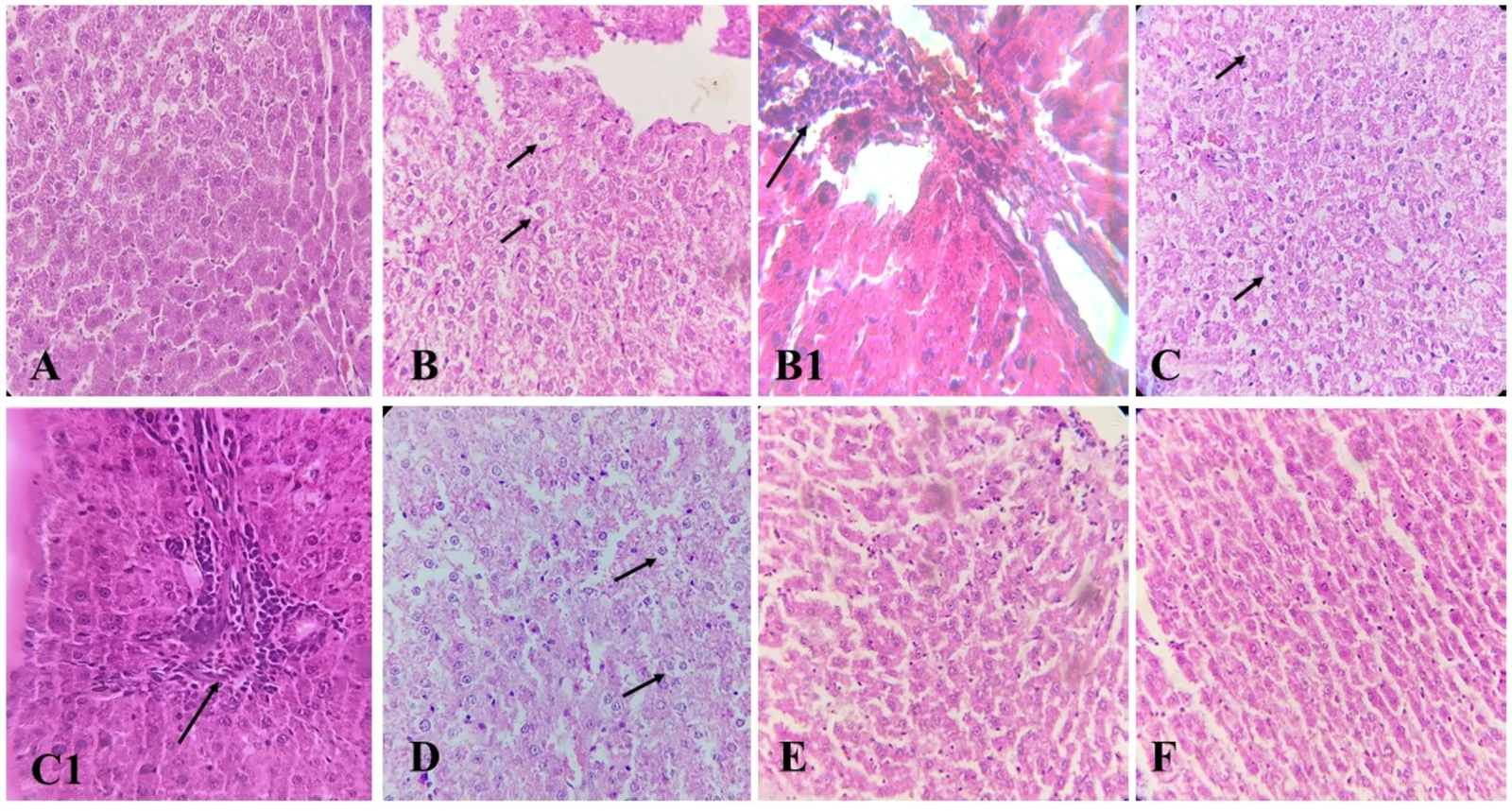
Histopathological effects of different doses of crocin and orlistat on liver tissue in the studied groups, based on H&E staining (400×). Experimental groups (n = 7 per group) were defined as follows: ND, normal diet (control group); HFD, high-fat diet (Fat) group; Cr10, Cr30, and Cr50, animals receiving crocin at doses of 10, 30, and 50 mg/kg body weight, respectively; Orl, animals treated with orlistat. Representative H&E-stained liver sections are shown for: A: normal diet; B, B1: HFD; C, C1: HFD + crocin 10 mg/kg; D: HFD + crocin 30 mg/kg; E: HFD + crocin 50 mg/kg and F: HFD + orlistat 10 mg/kg. Arrows in images B, C, and D indicate hepatocellular ballooning (foam cells), while arrows in images B1 and C1 highlight lymphocyte infiltration (inflammation).

**Table 2:** Semi-quantitative histopathological scoring of liver tissue in experimental groups.

Groups	Lipid accumulation	Hepatocellular ballooning	Inflammation (lymphocyte infiltration)	histopathological changes of liver
**Cont**	0	0	0	0
**Fat**	3	3	3	3
**Cr 10**	3	2	3	3
**Cr 30**	2	1	1	1
**Cr 50**	1	0	0	0
**Orl 10**	0	0	0	0

This table presents the effect of different doses of crocin on the histopathological features of the liver tissue. Histopathological changes were assessed in H&E-stained liver sections using a semi-quantitative scoring system for steatosis, inflammation, and ballooning. Steatosis was scored as 0 = negative, 1 = mild, 2 = moderate, and 3 = severe. Ballooning, Inflammation and histopathological changes were also scored according to severity. Cont: Normal Diet (ND), Fat: High-Fat Diet (HFD), Cr10: crocin 10 mg/kg, Cr30: crocin 30 mg/kg and Cr50: crocin 50 mg/kg, and Orl: orlistat.

## Discussion

The present study demonstrated that crocin exerts significant hepatoprotective effects in a HFD-induced model of NAFLD. Specifically, crocin administration led to a marked improvement in lipid profile parameters, including reductions in TG and LDL-C levels, alongside significant decreases in hepatic enzymes (ALT, AST, and ALP). In addition, crocin attenuated oxidative stress, as evidenced by decreased MDA and nitrite levels, and enhanced antioxidant mechanisms through increased SOD, CAT, and total thiol content. Histopathological findings further confirmed a reduction in hepatic steatosis and inflammation.

NAFLD, which is characterized by lipid accumulation in the liver, is the most common cause of liver diseases and encompasses several liver disorders, including steatosis, NASH, fibrosis, cirrhosis, and liver failure [43]. Although the etiology of NAFLD remains unclear, studies have demonstrated the roles of a HFD [44], obesity, hyperglycemia, liver damage [45], histological changes, altered functional enzymes in liver tissue [46], and oxidative stress in the development of NAFLD [47]. These factors adversely affect lipids, proteins, and DNA, contributing to the onset of various diseases [48].

The pathogenesis of NAFLD is a multifactorial process involving dysregulation of lipid metabolism, insulin resistance, oxidative stress, and chronic inflammation, with AMPK playing a central regulatory role in hepatic energy homeostasis [49]. Activation of AMPK suppresses hepatic lipogenesis by downregulating key transcription factors such as SREBP-1c and FAS, while promoting fatty acid β-oxidation through upregulation of PPARα and CPT1 [29]. Crocin has been shown to significantly activate AMPK signaling, leading to reduced hepatic triglyceride accumulation and improved metabolic profiles in experimental NAFLD models [29]. Furthermore, insulin resistance, a key driver of NAFLD progression, contributes to increased free fatty acid influx and hepatic lipid accumulation, whereas crocin improves insulin sensitivity through modulation of AMPK-dependent pathways such as CDK5/PPARγ signaling [50]. In addition to metabolic regulation, oxidative stress and mitochondrial dysfunction play critical roles in the progression from simple steatosis to NASH, primarily through excessive production of ROS and hepatocellular injury [51]. Crocin has been reported to exert potent antioxidant effects by reducing ROS generation and improving mitochondrial function, thereby restoring cellular energy balance and attenuating liver damage [51]. Moreover, chronic inflammation mediated by NF-κB signaling contributes significantly to NAFLD progression, and crocin has been shown to suppress NF-κB activation and reduce pro-inflammatory cytokines such as TNF-α and IL-6 [52]. Collectively, these findings suggest that crocin exerts hepatoprotective effects through a multi-target mechanism involving modulation of AMPK signaling, improvement of insulin resistance, attenuation of oxidative stress, and inhibition of inflammatory pathways, highlighting its therapeutic potential in NAFLD management [29,49].

In contrast, crocin, one of the main components of saffron [25], exhibits a wide range of biological activities, including antihyperlipidemic, antioxidant, anti-atherosclerotic [27], antidiabetic [53], anti-inflammatory [28], anti-obesity [29], as well as promoting fat cell differentiation and lipolysis [30]. Studies indicate that crocin potentially protects against liver damage and counteracts biochemical alterations by regulating the expression of genes associated with NAFLD and NASH [52]. Furthermore, crocin has been shown to regulate the AMPK signaling pathway, reducing liver fat accumulation and liver damage. AMPK is a critical regulator of cellular energy metabolism [30]. Activated AMPK inhibits ACC, a key enzyme in lipid biosynthesis, and suppresses the expression of SREBP-1, CEBPα, PPARγ, and FAS during adipogenesis. Additionally, AMPK activation enhances lipolysis, thereby reducing adipose tissue mass [30]. Overexpression of AMPK in the liver increases fatty acid beta-oxidation and the expression of fatty acid-related genes while decreasing the expression of genes involved in lipogenesis, triglyceride content, and hepatic steatosis [29].

In our study, the HFD group exhibited a significant increase in the mean serum levels of TG, TC, and LDL-C compared to the control group, while a high dose of crocin significantly reduced the mean levels of TG and LDL-C. These findings are consistent with previous studies demonstrating that crocin reduces cholesterol, triglyceride, LDL, and glucose levels in diabetic rats and increases HDL levels [54]. Similarly, Liang Sheng et al. reported that crocin administration to diabetic rats lowered triglyceride, cholesterol, LDL, and VLDL and elevated HDL levels [55]. Recent studies have also shown that crocin can decrease liver enzyme levels in HFD-fed rats [46], and Binnaz Elif Cosgun et al. reported that crocin reduces CCl4-induced liver enzyme elevations in rats [56]. The lipid-lowering effect of crocin in our study is therefore in close agreement with these previous reports.

Liver damage causes the leakage of certain aminotransferases from the cytosol into the bloodstream, which serves as an indicator of liver injury [57]. An increase in AST and ALT levels reflects damage to the liver plasma membrane and is considered a marker of liver damage, inflammation, and necrosis [58]. The protective effect of crocin against liver damage may be attributed to its potent antioxidant properties, which help maintain the integrity of liver cell membranes, reduce tissue injury, and prevent the release of cellular enzymes into the serum [57]. Our findings demonstrated that intraperitoneal injection of crocin at higher doses, as well as orlistat, significantly reduced AST and ALP levels compared to the HFD group. These results are in line with preclinical investigations showing that crocin significantly reduces hepatic triglyceride accumulation, improves serum transaminases, and attenuates steatosis through modulation of AMPK signaling, lipid metabolism, and oxidative stress pathways [2,59]. The consistency of our findings with these studies further supports the hepatoprotective role of crocin.

Orlistat is a well-established anti-obesity drug that primarily reduces dietary fat absorption through inhibition of gastrointestinal lipases; however, its effects on liver-specific outcomes in NAFLD are generally modest and inconsistent [60]. In contrast, crocin appears to exert multi-target intracellular effects, particularly through activation of AMPK, leading to suppression of hepatic lipogenesis and enhancement of fatty acid β-oxidation [61]. In addition, crocin improves insulin sensitivity and metabolic dysfunction, contributing to improved lipid profiles and reduced adiposity [53]. Furthermore, crocin exerts potent antioxidant and anti-inflammatory effects by reducing ROS and modulating inflammatory and apoptosis-related pathways [52,62], which are key drivers of NAFLD progression [59]. Our study demonstrated that crocin produced comparable or superior effects to orlistat on most parameters, suggesting that crocin may offer additional benefits through direct hepatic mechanisms.

Oxidative stress results from an imbalance between free radicals and antioxidant enzymes [63]. This imbalance causes detrimental changes in lipids, proteins, and DNA, leading to various diseases [48]. The liver is particularly vulnerable to ROS, and oxidative stress plays a significant role in the exacerbation and progression of liver diseases. Oxidative stress increases steatohepatitis and promotes cell death. The Fas receptor is known as an inducer of apoptosis, the primary mechanism of cell death [52]. Crocin, due to its strong antioxidant properties, scavenges free radicals such as superoxide anions and provides a protective effect against oxidative stress [64]. The Fas receptor activates caspase-3 gene expression in the extrinsic apoptosis pathway, which is stimulated by oxidative stress [65]. Crocin may exert anti-apoptotic effects by reducing the expression of Fas and caspase-3 genes, thereby mitigating liver disease progression by improving NASH and oxidative stress [52].

In our study, a HFD increased the levels of MDA and nitrite while decreasing the levels of SOD, CAT, and total thiol. Administration of crocin at higher doses, as well as orlistat, significantly decreased the levels of MDA and nitrite and increased the levels of SOD and CAT. Additionally, the highest dose of crocin and orlistat significantly reduced total thiol levels. These findings are in close agreement with several previous studies. Clinical investigations have reported that crocin improves systemic oxidative stress markers and reduces pro-inflammatory cytokines such as TNF-α and IL-6, which are key mediators of NAFLD progression [66]. Luo et al. demonstrated that crocin alleviates hepatic steatosis through activation of AMPK [29]. Activation of AMPK is known to inhibit lipogenesis via suppression of SREBP-1 and FAS, thereby reducing lipid accumulation in hepatocytes [61]. Several studies have reported that crocin causes reductions in oxidative stress markers, including MDA, along with increased activity of endogenous antioxidant enzymes such as SOD and CAT [66,67]. Yaribeygi et al. demonstrated that crocin potentiates antioxidant defense system and improves oxidative damage in liver tissue in diabetic rats [62]. Fathi Moghadam et al. also reported that crocin causes a reduction in MDA levels and an increase in SOD activity [68]. Additionally, Kalantar et al. indicated that methotrexate elevates MDA and NO levels and decreases CAT and SOD activities in liver tissue, while crocin treatment reversed these changes [69]. The consistency between our results and these studies confirms the antioxidant properties of crocin.

Histopathological investigations in the present study demonstrated that a HFD causes lipid accumulation, inflammation, ballooning, and abnormal liver tissue structure. In contrast, administration of crocin and orlistat prevented these changes. Previous studies have shown that fat accumulation increases the liver tissue’s susceptibility to other pathogenic factors, such as oxidative stress, which contributes to the progression from steatosis to steatohepatitis, fibrosis, and cirrhosis [70]. Regarding the relationship between oxidative stress and liver tissue inflammation [71], the present study confirms that HFD induces oxidative damage in the liver. This induction of oxidative stress is further supported by the increased levels of MDA and alterations in antioxidant enzyme activity in the livers of HFD-fed rats. Our histopathological findings are consistent with previous reports showing that crocin decreases inflammatory signaling and improves mitochondrial function, contributing to reduced hepatic injury and lipid deposition [72]. Moreover, crocin has been reported to modulate key inflammatory pathways, particularly through inhibition of NF-κB, leading to decreased production of pro-inflammatory cytokines, and this anti-inflammatory action likely contributes to the observed improvement in hepatic histology [72]. The anti-apoptotic effects of crocin also appear to play a role in its hepatoprotective function, and experimental evidence suggests that crocin can inhibit apoptosis via modulation of Fas receptor signaling and caspase pathways [73]. Additionally, recent studies have indicated that crocin may attenuate hepatic fibrosis by targeting pathways such as TGF-β and TIMP-1 [74]. Although these molecular mechanisms were not directly assessed in the present study, the observed biochemical and histological improvements are consistent with these reported pathways. Clinical evidence further supports the beneficial effects of crocin in metabolic and inflammatory conditions. Crocin supplementation has been shown to reduce inflammatory biomarkers in patients with type 2 diabetes mellitus and other chronic conditions. These human studies complement our experimental findings and support the translational potential of crocin for human NAFLD [75,76].

Overall, the current body of evidence suggests that crocin exerts multi-target hepatoprotective effects through coordinated regulation of AMPK-mediated lipid metabolism, attenuation of oxidative stress, and suppression of inflammatory pathways. Collectively, both experimental and clinical findings converge to support crocin as a promising therapeutic candidate for NAFLD. However, some limitations should be acknowledged. This study did not directly investigate the molecular signaling pathways underlying the observed effects, and therefore mechanistic conclusions remain inferential. Future studies incorporating gene and protein expression analyses are warranted to elucidate the precise pathways involved. Furthermore, large-scale, well-controlled clinical trials are needed to confirm its efficacy in human NAFLD populations.

## Conclusion

In conclusion, the findings of this study demonstrate the role of crocin as a multi-target compound with hypolipidemic, antioxidant, and anti-inflammatory properties and exerts protective effects by preventing the development of NAFLD. These effects collectively contribute to its hepatoprotective action and highlight its potential as a therapeutic candidate for NAFLD.
